# Growth and Nitrogen Uptake Characteristics Reveal Outbreak Mechanism of the Opportunistic Macroalga *Gracilaria tenuistipitata*

**DOI:** 10.1371/journal.pone.0108980

**Published:** 2014-10-09

**Authors:** Chao Wang, Anping Lei, Kai Zhou, Zhengyu Hu, Wenlong Hao, Junda Yang

**Affiliations:** 1 College of Life Sciences, Shenzhen University, Shenzhen, China; 2 Institute of Hydrobiology, Chinese Academy of Sciences, Wuhan, China; 3 Shenzhen Marine Environment and Resources Monitoring Center, Shenzhen, China; Texas A&M University at Galveston, United States of America

## Abstract

Macroalgae has bloomed in the brackish lake of Shenzhen Bay, China continuously from 2010 to 2014. *Gracilaria tenuistipitata* was identified as the causative macroalgal species. The aim of this study was to explore the outbreak mechanism of *G. tenuistipitata*, by studying the effects of salinity and nitrogen sources on growth, and the different nitrogen sources uptake characteristic. Our experimental design was based on environmental conditions observed in the bloom areas, and these main factors were simulated in the laboratory. Results showed that salinity 12 to 20 ‰ was suitable for *G. tenuistipitata* growth. When the nitrogen sources' (NH_4_^+^, NO_3_^−^) concentrations reached 40 µM or above, the growth rate of *G. tenuistipitata* was significantly higher. Algal biomass was higher (approximately 1.4 times) when cultured with NH_4_^+^ than that with NO_3_^−^ addition. Coincidentally, macroalgal bloom formed during times of moderate salinity (∼12 ‰) and high nitrogen conditions. The NH_4_^+^ and NO_3_^−^ uptake characteristic was studied to understand the potential mechanism of *G. tenuistipitata* bloom. NH_4_^+^ uptake was best described by a linear, rate-unsaturated response, with the slope decreasing with time intervals. In contrast, NO_3_^−^ uptake followed a rate-saturating mechanism best described by the Michaelis-Menten model, with kinetic parameters *V_max_* = 37.2 µM g^−1^ DM h^−1^ and *K_s_* = 61.5 µM. Further, based on the isotope ^15^N tracer method, we found that ^15^N from NH_4_^+^ accumulated faster and reached an atom% twice than that of ^15^N from NO_3_^−^, suggesting when both NH_4_^+^ and NO_3_^−^ were available, NH_4_^+^ was assimilated more rapidly. The results of the present study indicate that in the estuarine environment, the combination of moderate salinity with high ammonium may stimulate bloom formation.

## Introduction

Macroalgal blooms have a broad range of ecological impacts and are considered to be harmful algal blooms [1]. Many studies have addressed the potential ecological and environmental consequences of macroalgal blooms [2], [3], including uncoupled biogeochemical cycles, degraded intertidal environment, reduced biodiversity, hypoxia or anoxia, destruction of coastal marine habitats (e.g., seagrass) and economic losses of marine industries (e.g., fisheries and tourism) [4], [5], [6], [7], [8], [9].

**Figure 1:**
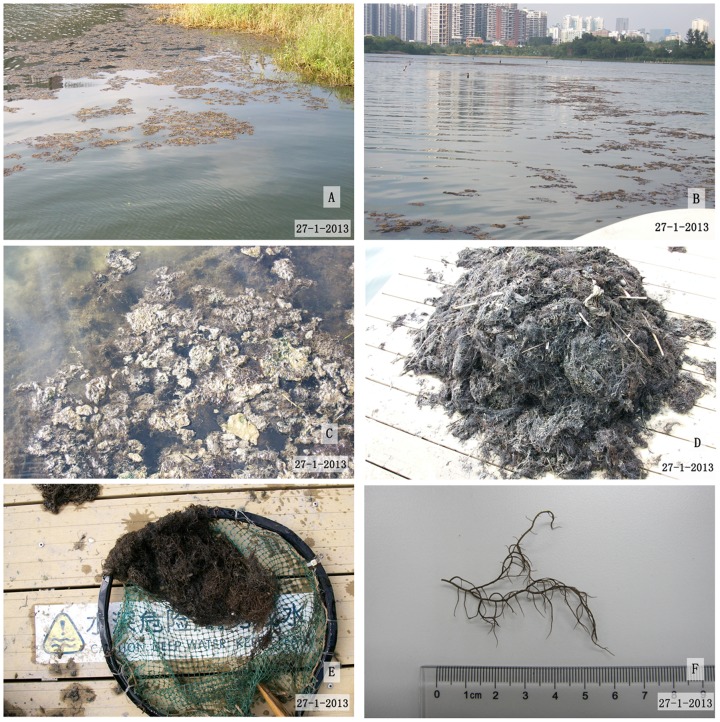
The *G. tenuistipitata* in OCT North Lake. A,B,C) The *G. tenuistipitata* bloom in OCT North Lake, D) The salvaged *G. tenuistipitata*, E) Samples of *G. tenuistipitata* collected, F) Observation and identification of macroalgae.

In January 2013, red algae bloomed in the OCT North Lake of Shenzhen. Initially, a bloom of macroalgae occurred near the shore (Fig. 1A), then the lake surface became full of floating red macroalgae (Fig. 1B). Just 1 month later, from the surface to the bottom of the lake were filled with this kind of fast growing macroalgae and decomposition happened afterwards (Fig. 1C and D), becoming a serious issue for the local administration. The over-grown red algae were identified as Rhodophyta, Gigartinales, Gracilariaceae, Gracilaria, *Gracilaria tenuistipitata* (Fig. 1E and F). The damage caused by the bloom of *G. tenuistipitata* during this period included tourism and cleanup costs (about $ 50,000) (according to the local management office). Besides this, along with macroalgae decomposition, dead fish were also observed, indicating the lake ecological system has been damaged, to some extent.

Several hypotheses were proposed to explain the occurrence of macroalgal blooms in estuarine areas [10], [11], [12]. The OCT North Lake is connected to Shenzhen Bay which encountered strong anthropogenic influence, there are ∼0.2 million tons/day land-based fresh wastewater input [13], resulting in Shenzhen Bay as perennial brackish water (salinity 8–20‰) with high nitrogen loadings (essentially NH_4_^+^) leading to eutrophication [14]. Considering the estuarine environment, the effect of salinity on growth of *G. tenuistipitata* was investigated in this study. The ability to uptake and assimilate nutrients rapidly is one of the characteristics of opportunistic species. Concerning mechanisms of algal blooms, the bioavailability of nitrogen is the most important among all of the biogenic elements [2], [3]. In coastal areas, nitrogen is available to macroalgae majorly in the forms Dissolved Inorganic Nitrogen (DIN): ammonium (NH_4_^+^) and nitrate (NO_3_^−^). It is well known that fast growing species are positively affected by increased nutrient availability [15], thus, the effects of NH_4_^+^ and NO_3_^−^ on macroalgal growth were also investigated in this study.

A profound knowledge of the nitrogen uptake kinetics of *G. tenuistipitata* is important essentially for two reasons: It helps to study the algal potential assimilation efficiency and it is fundamental to assess its potential as a biofilter in estuaries and coastal waters [16], [17], [18]. There is a set of works studying the uptake kinetics in Marine macroalgae Chlorophyta and Rhodophyta species [19], [20], [21], [22]. The Michaelis-Menten equation was generally used to describe nutrient uptake by phytoplankton over the past few years [23], while the results varied depending on algal species and nutrients. Some macroalgae followed Michaelis-Menten kinetics, some did not, and some species did not show a clear way of assimilating NH_4_^+^ or NO_3_^−^ [22]. Our work intended to provide more information regarding the nitrogen uptake dynamics of brackish water grown Rhodophyta species *G. tenuistipitata* in an attempt to elucidate the mechanism behind bloom formation. Short-term kinetics of NH_4_^+^ and NO_3_^−^ uptake were studied for the uptake characteristic of *G. tenuistipitata*. Nitrogen uptake has been studied for a variety of macroalgae, but the N species preference is still discussed [24], [25]. Elucidating the uptake strategy of *G. tenuistipitata* is essential because this red alga is the major bloom-forming species in nutrient-rich estuaries [26], [27]. One mechanism that may enhance bloom ability is the capability to choose and use different DIN species at the same time [19]. This isotope ^15^N tracer study was used to quantify uptake and assimilation strategy of NH_4_^+^ and NO_3_^−^ offered simultaneously in order to assess potential mechanisms that may enhance the bloom ability of *G. tenuistipitata* during irregular pulses of nitrogen sources.

## Materials and Methods

### Ethics Statement

Permission to investigation, public photos and sampling inside the OCT North Lake was approved through permits from the Overseas Chinese Town Holdings Company(OCT Group)and the Shenzhen government. Our field investigation did not involve endangered or protected species.

### Algal material

During the macroalgal bloom, thalli of *G. tenuistipitata* were sampled from OCT North Lake (22°31′46.06″N, 113°58′40.32″E), Shenzhen, in January 2013. Healthy algal thalli collected were transported to the lab in a cool box within 4 h. Then they were flushed several times with artificial seawater to remove epiphytes, slime, sediment and small grazers, and then cultured in nutrient-deplete sterile artificial seawater [28] with vitamins and trace elements added according to the f/2 medium at salinity 12±1‰. Each algal sample was cultured for 3–5 days with bubbling air before the experiment. The cultures were kept at the salinity 12±1‰, temperature 25±0.5°C, with irradiance of 56 µE m^−2^ S^−1^ and photoperiod of 12∶12 (Light: Dark).

### Salinity effects on growth rates

To determine the variable salinity effects on growth of *G. tenuistipitata*, we evaluated the Relative Growth Rates (RGR) of *G. tenuistipitata* at salinities of 4, 8, 12, 16, 20, 24, 28 and 32 ‰ over a 15-day period. Approximately 0.1 g wet weight of *G. tenuistipitata* was added to 600 mL sterile f/2 enriched artificial seawater in a 1-L glass beaker at conditions described above. Each algal sample was assigned at random. The enriched medium was renewed every four days. Four replicates were used for each treatment. Every 4 days, the *G. tenuistipitata* thalli were blotted dry and the fresh weight determined, and RGR were calculated: RGR = (ln *w_2_*-ln *w_1_*)/Δt, where *w_1_* is the initial fresh mass and *w_2_* is the fresh mass after Δt days.

### Nitrogen source effects on growth rates

The effects of nitrogen source on growth rates of *G. tenuistipitata* were tested for 15 days. For the nitrogen-effect experiment, two nitrogen sources, ammonium (NH_4_Cl) and nitrate (NaNO_3_) were applied. Nitrogen (NH_4_^+^ or NO_3_^−^) concentrations were adjusted to 5, 10, 20, 40, 60, 80, 100 µM, treatments without nitrogen addition were taken as control groups. 30 µM phosphate (KH_2_PO_4_) was added to all treatments to avoid P limitation [29]. The fresh algal thalli of 0.1 g was randomly put in a 1-L glass beaker filled with 600 mL nutrient-deplete sterile artifical seawater at conditions described above. All glass material used during the experiment had been acid washed with 10% HCL to prevent possible nutrient contamination. Nutrient-enriched media were renewed every four days. Four replicates were used for each treatment.

### Nitrogen source uptake

The nitrogen (NO_3_^−^ and NH_4_^+^) uptake of *G. tenuistipitata* was determined by monitoring the nutrient depletion of the medium. The nutrient-deplete experimental artificial seawater was obtained from *G. tenuistipitata* culture medium mentioned above. After cultured alga for 5 days then NH_4_^+^ and NO_3_^−^ concentrations were monitored daily until their concentrations were below the detection limit (0.05 µM N L^−1^), then culture media were filtered (GF/C) used as nutrient-depleted seawater medium. The uptake experiment was performed in 400 mL medium with nutrients added from stock solutions in 500-mL sterilized glass beakers. Uptake experiments with NO_3_^−^ or NH_4_^+^ were started with 10, 20, 40, 60, 80, 100 µM respectively. To avoid P shortage, 30 µM PO_4_^3−^ (KH_2_PO_4_) was added in the experiments. In order to determine the initial N concentrations, 2 ml seawater samples were taken before adding the macroalga. Further 2 ml seawater samples were collected at each time interval: 10, 20, 30 and 60 min. Seawater samples from both experiments were filtered (GF/C) and kept at −20°C until analysis (within 1 week). Analysis of NH_4_^+^ and NO_3_^−^ was carried out using a CleverChem 200 automated discrete analyzer (DeChem-Tech. GmbH, Germany) according to the manufacturer's instructions. Algal material was dried to a constant weight (48 h) in an oven kept at 60°C for dry weight. Uptake rates (*V*) were calculated from changes in substrate concentrations during each sampling interval using the equation: *V* =  [(*C_0_*×*V_0_*) − (*C_t_*×*V_t_*)]/(*t*×DM), where *C_0_* and *C_t_* are the substrate nutrient concentration, and *V_0_* and *V_t_* are the volumes before and after a sampling period (*t*), and DM is algal dry biomass.

For estimation of the kinetic parameters of each N source, uptake rates were plotted together in *V* vs *S* curves. The *V* (uptake rate) against *V*/*S* (*S* = NH_4_^+^ or NO_3_^−^ concentration) linear transformation of the Michaelis-Menten equation was used to determine whether the data could be described by Michaelis-Menten kinetics [30]. Data which showed saturation kinetics were fitted to the Michaelis-Menten equation: *V* =  (*V_max_*×*C*)/(*Ks* +*C*), using enzyme kinetics module (non-linear regression model) to analyze each replicate dataset according to the instructions of SigmaPlot 12.5 (SPSS Inc., Chicago, USA). The Michaelis–Menten equation and parameters, *V_max_* (maximum uptake rate) and *Ks* (substrate concentration at which uptake proceeds at half the maximum rate) were obtained from the analysis.

### Nitrogen preferences

After nitrogen starvation of 4–5 days, nitrogen uptake was estimated using stable isotopes as tracers. Uptake experiments were initiated by adding tracer amounts (50 µM N with 10 atom % ^15^N) of highly enriched (96–99%) ^15^N-labeled NO_3_^−^ or NH_4_^+^ to nutrient-deplete artificial seawater mentioned above placed in 30 ml acid-washed glass tubes. NO_3_^−^ or NH_4_^+^ were added in different ratios, with only one DIN species labeled with ^15^N per trial. After 1 h or 24 h, the algal thalli were briefly rinsed with deionized water, and dried at 60°C to constant weight (48 h), powdered with a Spex mixer (Spex CertiPrep, Inc., Edison, NJ), and analyzed for TN and ^15^N enrichment with an isotope ratio mass spectrometer (Europa Scientific Integra). Water samples were filtered (GF/C) and immediately analyzed for ammonium, nitrite, and nitrate. ^15^N enrichment was calculated based on the methods of Naldi and Wheeler [25].

### Data analysis

Separate one-way ANOVA were used to analyze the responses of *G. tenuistipitata* to the different salinity treatments or nutrient (NO_3_^−^ and NH_4_^+^) sources in terms of RGR. Data were initially examined for homogeneity of variance and *F*-tests were performed to test the significance of difference among treatments. Tukey test (equal variances) was then used to test the significance of difference between two specific treatments, if significant difference was found among treatments. Statistical analyses were performed with SPSS 18.0 (SPSS Inc., Chicago, USA).

## Results

### Effects of salinity and nitrogen sources on growth rate

**Figure 2:**
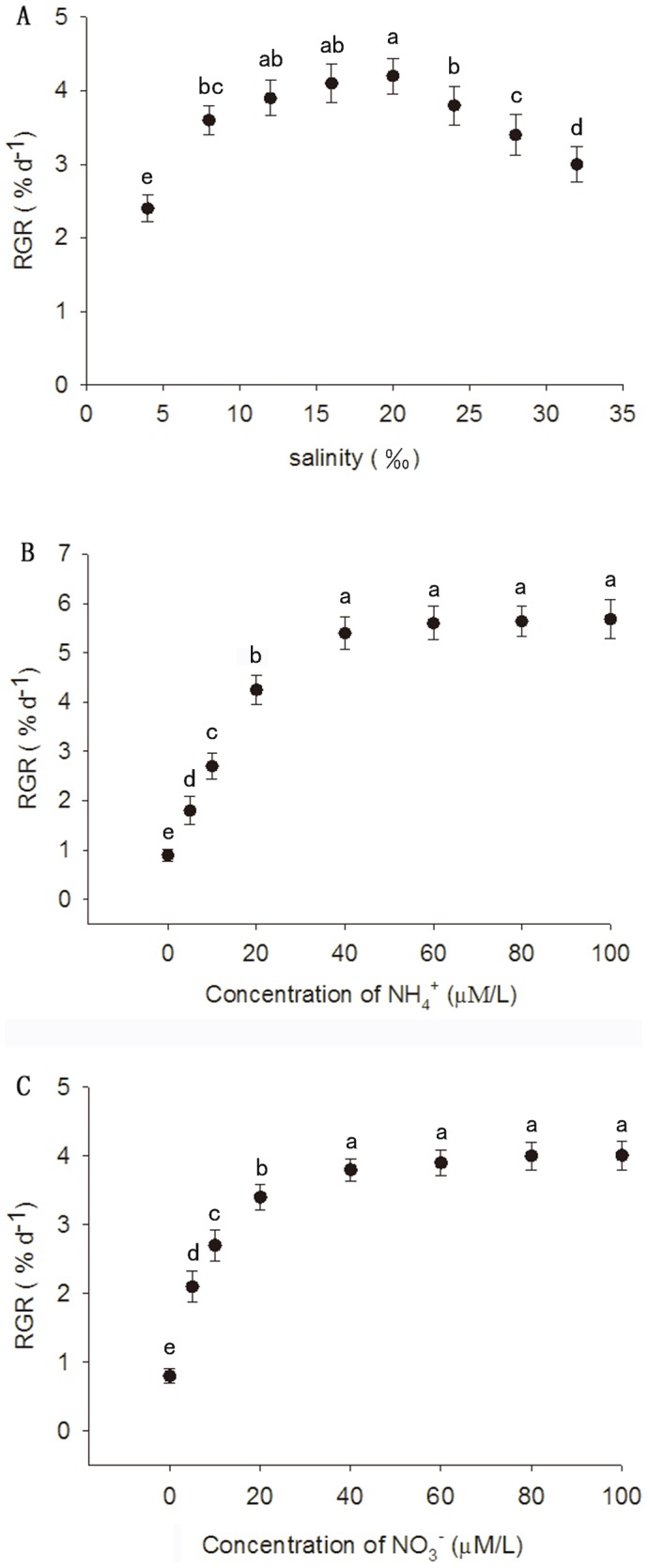
Effects of A) salinity, B) ammonium (NH_4_^+^) and C) nitrate (NO_3_^−^) on relative growth rate (RGR) of *G. tenuistipitata* after the 15-day culture. A) f/2 medium was enriched, B and C) Phosphate was enriched in all treatments (30 µM). Means and SD are shown (n = 4).

After the 15-day culture, there were significant differences in the RGR associated with salinity (One-way ANOVA, P<0.01). RGR of *G. tenuistipitata* was relatively high with salinity ranging from 12 to 20 ‰, and the highest RGR was 4.2%/d at salinity 20 ‰. Biomasses significantly decreased when cultured in low (4 ‰) (Tukey's test, P<0.05) or high (>20‰) salinity seawater (Tukey's test, P<0.05) (Fig. 2A).

There were significant differences between RGR of *G. tenuistipitata* associated with nitrogen addition (NH_4_^+^ or NO_3_^−^) after 15 days (One-way ANOVA, P<0.01). RGR was enhanced with increasing nitrogen (NH_4_^+^ or NO_3_^−^) concentrations, when the nitrogen (NH_4_^+^, NO_3_^−^) was up to 40 µM, however, there was no significant difference in RGR with higher concentrations (40–100 µM) (P>0.05, Fig. 2B and C), with the highest RGR observed in treatments of 100 µM nitrogen (NH_4_^+^ or NO_3_^−^). Interestingly, the algal biomass was higher (approximately 1.5 times) in algae cultured with NH_4_^+^ addition than that with NO_3_^−^ addition, respectively (Fig. 2B and C).

### Nitrogen (NH_4_^+^or NO_3_^−^) uptake

**Figure 3:**
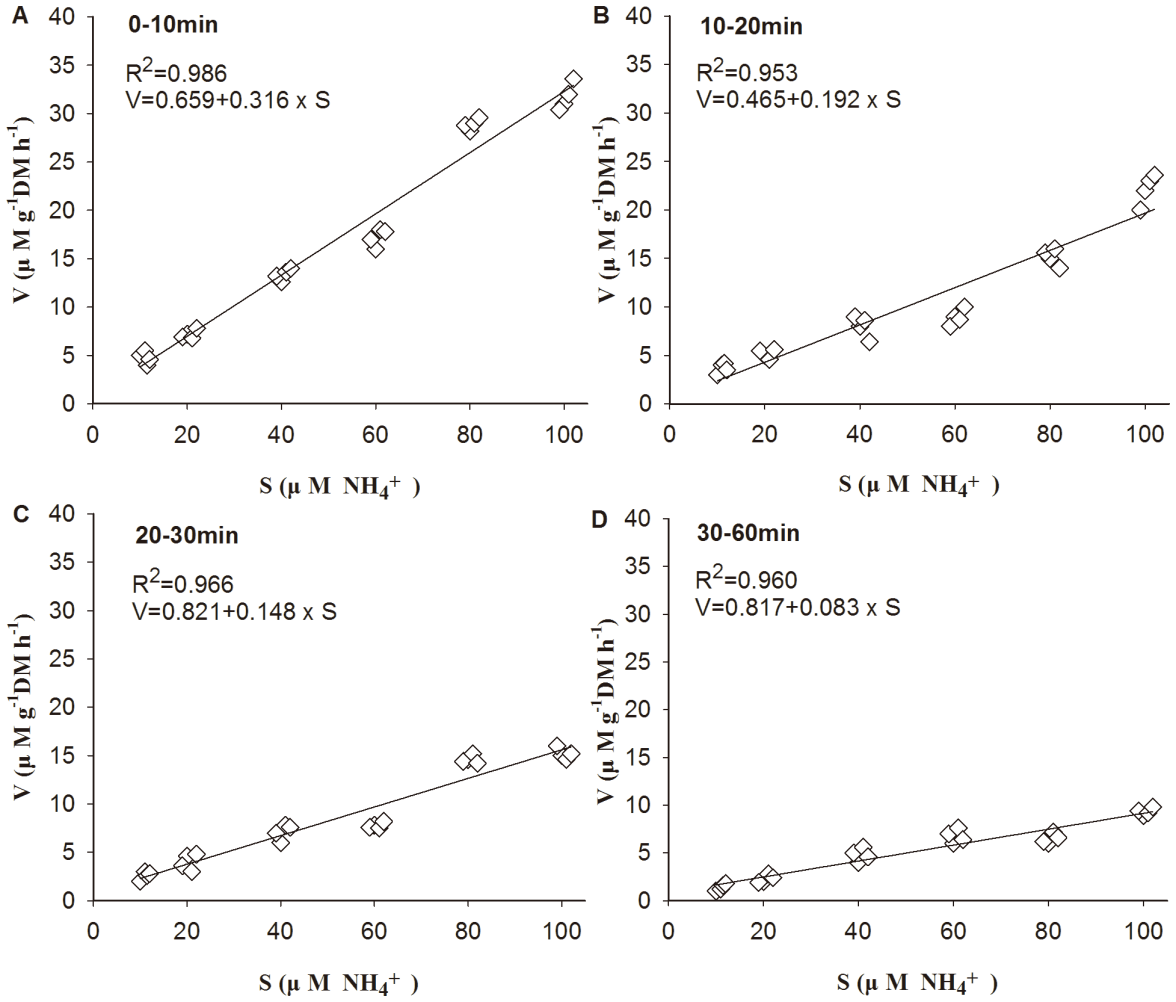
Ammonium (NH_4_^+^) uptake rates by *G. tenuistipitata* at different time intervals and different substrate concentration. Phosphate (30 µM) was added in all treatments.

During the 1 h experiment, uptake rates (*V*_0–1 h_) of *G. tenuistipitata* increased with NH_4_^+^ concentration levels. Uptake efficiency decreased, as suggested by a gradual decline in the uptake rates of NH_4_^+^ over sequential time intervals. The *V*-*S* of NH_4_^+^ was linearly positive correlated, best described by rate-unsaturated model (Fig. 3A, B, C and D). The bloom-forming red alga showed the highest uptake rates about 32 µM g^−1^DM h^−1^ for the first 10 min at the concentration of 100 µM.

**Figure 4:**
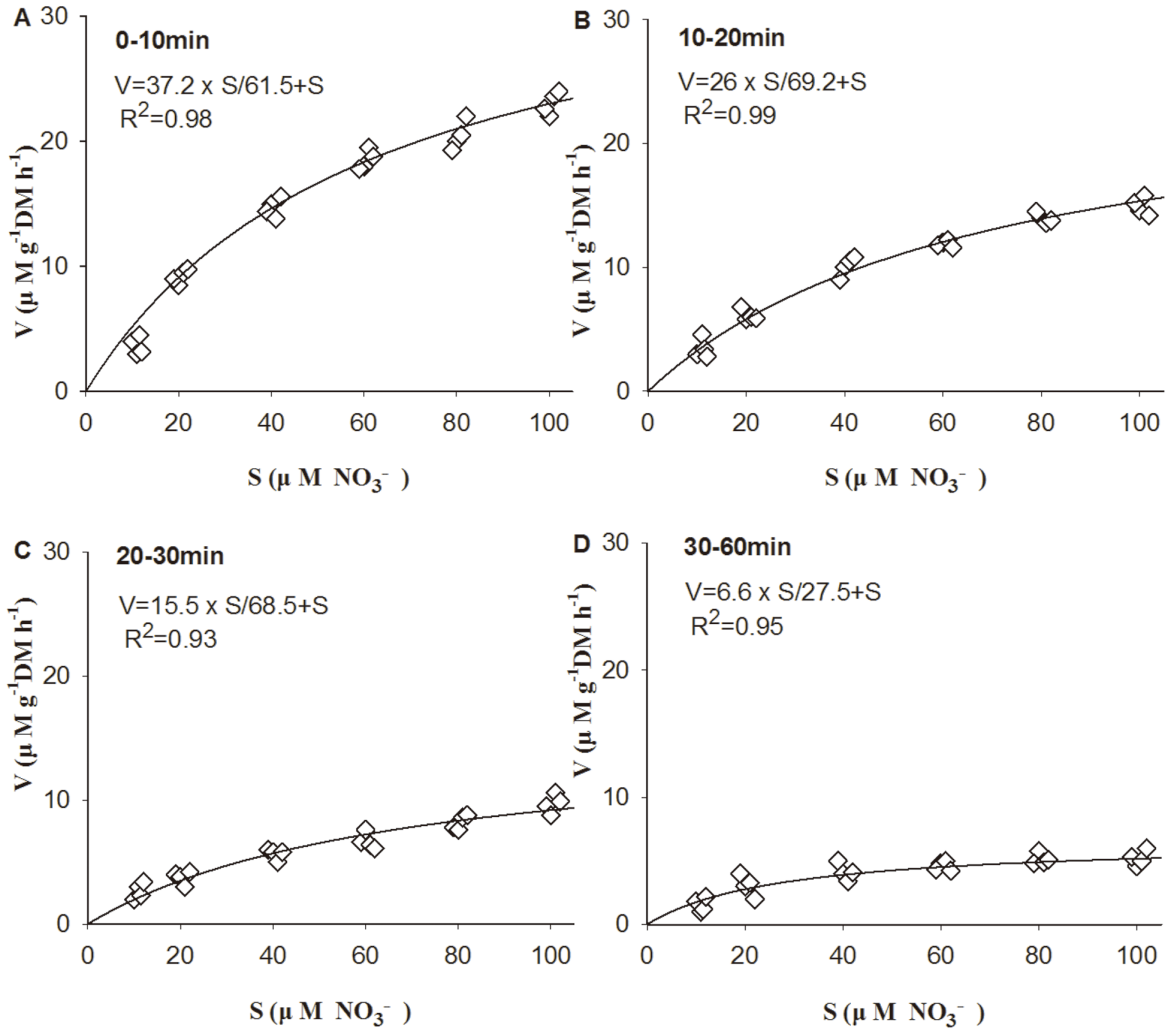
Nitrate (NO_3_^−^) uptake rates by *G. tenuistipitata* at different time intervals and different substrate concentration. Phosphate (30 µM) was added in all treatments.

High NO_3_^−^ uptake rates (*V*) of *G. tenuistipitata* were obtained from 0 to 10 min. Uptake rates for the NO_3_^−^ decreased over the time intervals. With increasing concentrations of NO_3_^−^, the uptake rates showed a tendency to saturation (Fig. 4A, B, C and D), and the *V*-*S* of NO_3_^−^ was fitted to Michaelis-Menten model. Kinetic parameters (*V_max_* and *K_s_*) following the Michaelis-Menten equation for NO_3_^−^ uptake by *G. tenuistipitata* at different time intervals during the 1 h experiment were shown in Table 1. The highest *V_max_* and *K_s_* values in the experiment with NO_3_^−^ were 37.2 µ M g^−1^ DM h^−1^ and 61.5 µM.

**Table 1:** Kinetic parameters (*V_max_* and *K_s_*) of the Michaels-Menten equation obtained from the rates for uptake of Nitrate (NO_3_^−^) by *G. tenuistipitata* at different time intervals.

	Time interval (min)	*V_max_* (SE)(µM g^−1^ DM h^−1^)	*K_s_*(SE)(µM)	R^2^
Nitrate (NO_3_^−^)	0–10	37.2 (1.85)	61.5 (6.26)	0.98
	10–20	26 (0.97)	69.2 (5.02)	0.99
	20–30	15.5 (0.2)	68.5 (1.85)	0.93
	30–60	6.6 (0.25)	27.5 (3.07)	0.95

### Nitrogen uptake preferences

**Figure 5:**
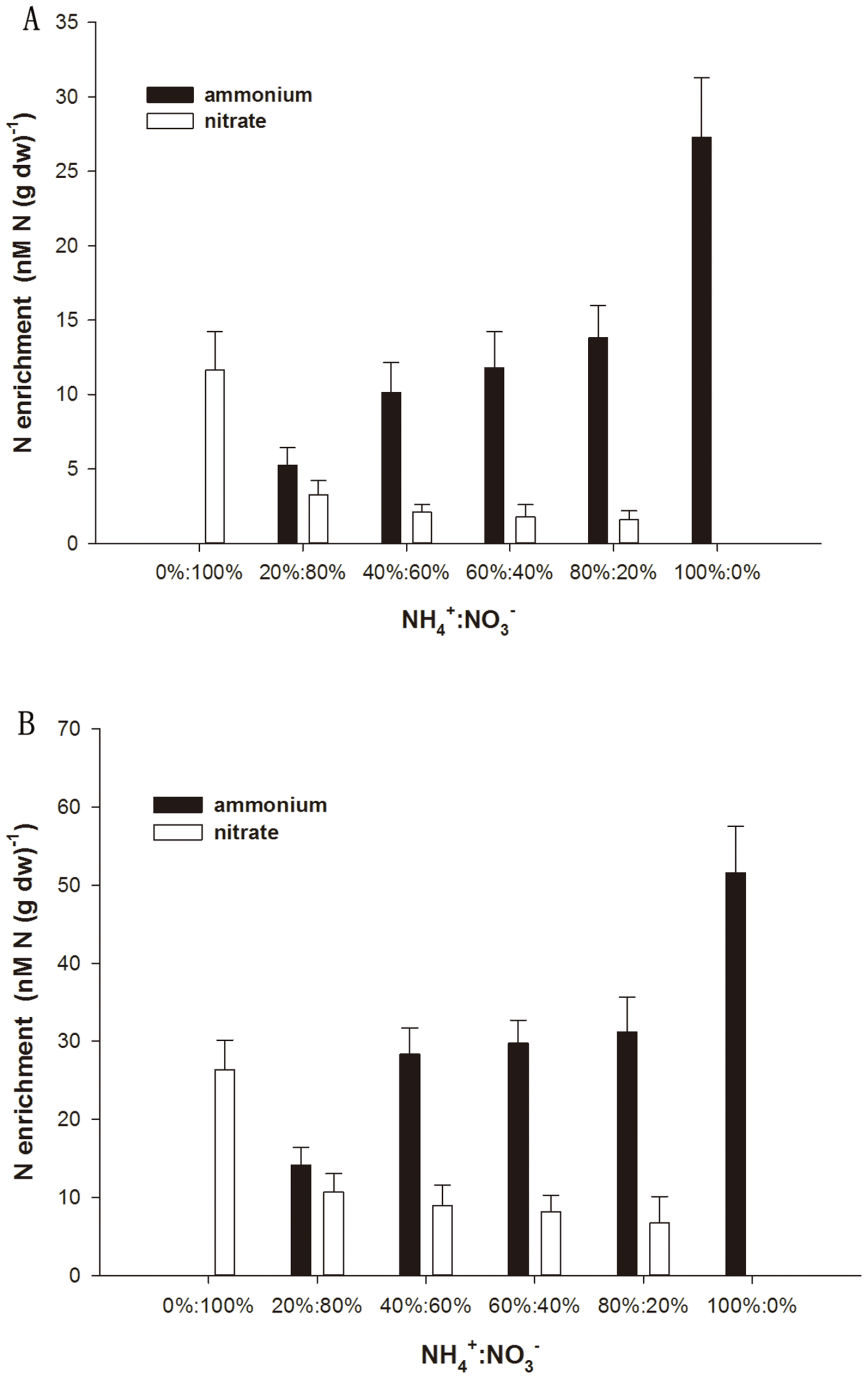
Ammonium (NH_4_^+^) and nitrate (NO_3_^−^) enrichment by *G. tenuistipitata* at different time intervals. A) 1 h and B) 24 h. The culture media contained 50 µM total N, but different proportions of NH_4_^+^: NO_3_^−^. Phosphate (10 µM) was added in all treatments.

The accumulation of ^15^N in *G. tenuistipitata* was rapid in 1 h, but decreased over time (Fig. 5A and B). ^15^N accumulation from sole source N cultures of NH_4_^+^ increased faster and during 24 h time interval reached an enrichment twice that for ^15^N of NO_3_^−^ (51.52 vs. 26.32 N enrichment, Fig. 5B). ^15^NH_4_^+^ accumulation by *G. tenuistipitata* exceeded ^15^NO_3_^−^ enrichment in all the two sources N cultures, regardless of the ratio of NH_4_^+^ versus NO_3_^−^ supplied and time. When the NH_4_^+^ supply was lower than that of NO_3_^−^ (20%:80%, 40%:60%), more ^15^NH_4_^+^ was accumulated. These results revealed that when NH_4_^+^ and NO_3_^−^ were supplied simultaneously, *G. tenuistipitata* preferentially assimilate NH_4_^+^.

## Discussion

Salinity in the estuarine or coastal areas is a reflection of the balance of land source flows and seawater from tidal flux. High tide and water exchange have changed the balance of freshwater and seawater and caused a change of salinity within the OCT North Lake. Salinity is an important influence on the growth, photosynthesis, and respiration of *Gracilaria* spp. [31], [32]. Bunsom found that salinity was the most crucial factor affecting *G. tenuistipitata* growth and the highest growth rate was at 25‰ [33], and it can tolerate salinities as higher as 39‰ [31]. In this study, the highest growth rate of *G. tenuistipitata* was at 20‰, instead of 25‰, perhaps due to perennial exposure to brackish water. Generally, lower salinities often inhibit growth of seaweeds, affect branching patterns and promote changes in their chemical composition [34], [35]. Our results showed the lowest growth rate was at 4‰, which was consistent with the result of low salinity growth inhibition. This study revealed that *G. tenuistipitata* from Lake Shenzhen grew well across a wide range of salinities (4–32‰). At the bloom period in January 2013, the salinity was 12.6‰, which was in the favorable range of this macroalga. Adaptability for low salinity might be one of the mechanisms of macroalgal bloom.

Nitrogen is one of the most important limiting factors for phytoplankton growth in aquatic systems [15], [36]. NH_4_^+^ and NO_3_^−^ are the principal forms of DIN in estuarine environments where nutrient supply is variable and unpredictable [37], [38]. NH_4_^+^ is primarily supplied to estuaries by sewage spills and treated wastewater [39]; NO_3_^−^ is the predominant source in riverine [40] and groundwater inputs [41]. The algae of the genus *Gracilaria* are characterized by a simple thallus with a high surface area to volume ratio, allowing for a rapid and efficient response to nutrient input, resulting in high rates of nutrient uptake and growth [15], [42]. Our studies reported the maximum average RGR of *G. tenuistipitata* was 5.68%/d at 100 µM NH_4_^+^ and 4.01%/d at 100 µM NO_3_^−^, compared with an RGR in control groups of less than 0.9%/d. The growth of *G. tenuistipitata* became faster with increased DIN (NH_4_^+^or NO_3_^−^) concentration. Nutrient enrichment, due to high N inputs via anthropogenic injection and regenerative processes, has contributed to enhanced growth of opportunistic macroalgae [2]. During the bloom period, in the OCT North Lake, nitrogen in shallow waters is often in the form of NH_4_^+^ with average concentration of 60 µM, and NO_3_^−^ of 35 µM, P (PO_4_^3−^) of 5 µM. DIN concentration was in the range of significantly positive stimulating this alga growth, while P could not meet this algal need according to the Redfield N (16): P (1) ratio, indicating that P was limiting during the bloom.

Opportunistic macroalgae, often have higher nitrogen uptake rates than other more specialized algae [43]. A high uptake rate allows these opportunistic macroalgae to use nutrients efficiently [44]. With respect to uptake rate, some species followed the classic Michaelis-Menten kinetics for N uptake [22], [23], while others have exhibited sustained linear, rate-unsaturated uptake mainly due to eutrophic/oligotrophic level [45], [46], [22], [48], [49]. Normally, NO_3_^−^ uptake shows saturation kinetics [47]. Smit [48] investigated nitrogen uptake by *Gracilaria gracilis* and concluded that NO_3_^−^ uptake followed a rate-saturating mechanism was best described by the Michaelis-Menten model. A set of studies have used extraordinarily high NH_4_^+^ concentration (500 µM above levels) as Table 2 shows, which is toxic to many marine organisms [50], but the results were still positively accorded with Michaelis-Menten or linear models. The max DIN concentration (100 µM) in this study was based on the in situ environmental concentrations, and the principal finding about this long-term brackish water lived macroalga for the first time that the NH_4_^+^ uptake of *G. tenuistipitata* was linear, while the uptake kinetics of NO_3_^−^ were distinctly different from those of NH_4_^+^. The uptake rate of NO_3_^−^ over time fitted Michaelis-Menten kinetics. This indicated high uptake rate and affinity capability for NH_4_^+^. During the bloom, NH_4_^+^ was abundant in OCT North Lake, which might be another reason for macroalgal bloomed.

**Table 2:** Comparison of *V_max_* (µM g^−1^ DM h^−1^), *K_S_* (µM) values and substrate range (µM) for several Chlorophyta and Rhodophyta species in the marine or estuarine environment.

	Species		NH^4+^			NO^3−^		Reference
		*V_max_*	*K_s_*	S. range	*V_max_*	*K_s_*	S. range	
*Chloro-phyta*	*Ulva lactuca*	450	85	∼1200	116	34	∼50	[20]
	*Ulva prolifera*	284.6	25.1	∼250	124.3	15.1	∼250	[21]
	*Ulva linza*	250.2	37	∼250	109.1	23	∼250	[21]
	*Cladophora montagneana*	130	20.7	∼357	42.1	1.4	∼357	[56]
*Rhodo-phyta*	*Gracilaria foliifera*	23.8	1.6	∼15	9.7	2.5	∼15	[55]
	*Gracilaria pacifica*	21.5	50.9	∼15	6.0	26.8	∼30	[19]
	*Gracilaria gracilis*	—	—	∼40	35	5.6	∼50	[48]
	*Gracilaria vermiulophylla*	—	—	∼160	+	+	∼400	[22]
	*Catenella nipae*	550	692	∼1200	8.3	5	∼50	[20]
	*Gracilaria tenuistipitata*	—	—	∼500	*	*		[49]
	*Gracilaria tenuistipitata*	—	—	∼100	37.2	61.5	∼100	Present study

— means linear, rate-unsaturated response.

+ means not clear.

*means no report.

Generally, nitrogen uptake in macroalgae depends on intracellular levels [15] and environmental nitrogen concentrations [51]. Biological factors such as metabolism, morphology, tissue type, the age of the algae, and nutritional history [42], [52], [53], all influence nitrogen uptake by macroalgae. The DIN uptake pattern can be explained by different nitrogen assimilation mechanisms. The process of nitrogen uptake and assimilation in macroalgae involves transport from the water across the cell membrane and then assimilation into organic compounds, followed by incorporation into proteins and macromolecules for growth [40]. For NO_3_^−^, there is the additional step of reduction to NH_4_^+^ by nitrate reductase after uptake [54]. One explanation for NH_4_^+^ affinity may be that energy required for nitrate reduction could be saved [53].

Two parameters of Michaelis-Menten kinetics, namely *V_max_* and *K_s_*, contain ecologically relevant information relating to the nutrient uptake ability of a species under conditions of varying nutrient availability. In this study, the *V_max_* was 37.2 and *K_s_* was 61.5 for uptake of NO_3_^−^ by *G. tenuistipitata*in in Table 1. While, the *V_max_* declined over time intervals, indicating initially very high uptake rates and lower sustained uptake rates. Previous work addressed the uptake differences between Chlorophyta and Rhodophyta species in the marine or estuarine environment [19], [20], [21], [22], [48], [49], [55], [56]. Commonly, Chlorophyta follow Michaelis-Menten kinetics for NH_4_^+^ and NO_3_^−^ [20], [21], [56], Rhodophyta differ depending on the species [19], [22], [48]. Higher *V_max_* were found in Chlorophyta for NO_3_^−^ [20], [21]. *G. tenuistipitata* has the relatively higher *V_max_* in Rhodophyta species, implying higher assimilating and storing capability for NO_3_^−^. However, the *V_max_/K_s_* value was 0.6, less than 1, indicating the competitive advantage for NO_3_^−^ is not so obvious compared with other Rhodophyta species. During the bloom, NO_3_^−^ concentration was relatively low, while NH_4_^+^ was abundant. What's more, resilience to low salinities might be a principal reason for bloom of *G. tenuistipitata*.

Environmental factors, including temperature, salinity, nutrient levels and other relevant competitors, are principally responsible for regulating macroalgal bloom occurrence [6], [7]. Because Shenzhen Bay is in the subtropical zone, the water temperature is relatively constant between day and night, without great changes between different months, so our research did not focus on its influence on algal RGR, with attention to the effect of salinity and DIN. Besides, the competition for resources (especially nutrients) is ubiquitous in marine phytoplankton [55], and the chemical defense system is another important potential mechanism for algal competition. Allelopathy, one type of chemical defense competition between plants, can be a potent influential factor. Xu et al. [57] found allelopathic interactions happened between green tide-forming species *Ulva prolifera* and macroalga *Gracilaria lichvoides*, indicating that some allelochemicals released by both of the macroalgae could account for the physiological inhibition of growth. In our study, there is only one species, namely *G. tenuistipitata*, lived and bloomed in the OCT North Lake until 2014, no other macroalga was found. So we neglect the competing role among macroalgae.

From our ^15^N tracer study, we found the opportunistic macroalga *G. tenuistipitata* actively utilizes multiple DIN species simultaneously, and prefers the most energetically efficient (NH_4_^+^) when available. Other previous reports have also found NH_4_^+^ uptake preference. D'Elia and DeBoer found the priority of NH_4_^+^ uptake, even in very low concentrations (∼5 µM), and its presence seriously deceased the uptake of NO_3_^−^ by *Gracilaria foliifera* (Forsskal) *Borgesen* [58], while Hanisak and Harlin determined that only 1 µM NH_4_^+^ was enough to decrease NO_3_^−^ uptake in *Codium fragile* (van Goor) *Silva* [59]. Haines and Wheeler showed that NO_3_^−^ uptake by *Hypnea musciformis* (Wulfen) *Lamouroux* was reduced by 50% in the presence of about 18 µM NH_4_^+^ [60]. Our study also found a decrease of NO_3_^−^ uptake when NH_4_^+^ was present. The result suggested that when NH_4_^+^ and NO_3_^−^ were supplied in equal or less ratio concentrations simultaneously, more NH_4_^+^ was taken up and assimilated.

From the present study, three key conclusions were obtained: *i*) the growth of *G. tenuistipitata* was regulated by salinity and N availability in environment. Its tolerance of relatively low salinity in estuarine environment seems to be a principal factor for its bloom rather than the other species. Its high growth rate and extraordinary capabilities for utilizing nutrients, like DIN likely contribute to macroalgal outbreaks; *ii*) The DIN: NH_4_^+^ and NO_3_^−^ uptake kinetics of *G. tenuistipitata* are different. NH_4_^+^ uptake demonstrated a rate-unsaturated response, but NO_3_^−^ uptake followed a rate-saturating mechanism which fit the Michaelis-Menten model, with kinetic parameters *V_max_* (37.2 µ M g^−1^ DM h^−1^) and *K_s_* (61.5 µ M), indicating a higher uptake efficiency of NH_4_^+^ than NO_3_^−^; *iii*) The strategy of *G. tenuistipitata* assimilating different forms of DIN showed that NH_4_^+^ incorporated faster than NO_3_^−^. We propose that when NH_4_^+^ and NO_3_^−^ were supplied simultaneously, *G. tenuistipitata* preferentially assimilated NH_4_^+^. One mechanism that may enhance bloom ability is the strategy to choose and use different DIN in estuarine environments.
